# Supplementary material for: The RNA secondary structure of androgen receptor-FL and V7 transcripts reveals novel regulatory regions
Source: Nucleic Acids Res. 2024 Mar 30;52(11):6596–613. doi: 10.1093/nar/gkae220 (PMC11194067; doi:10.1093/nar/gkae220)

AR_M1_-1_5U_1

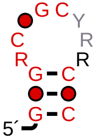

AR_M2_-1_5U_1

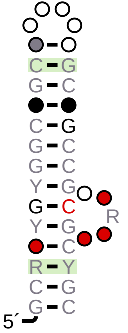

AR_M3_-1_5U_1

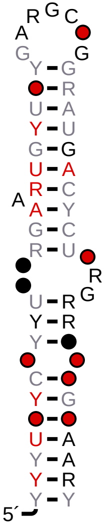

AR_M4_-1_5U_1

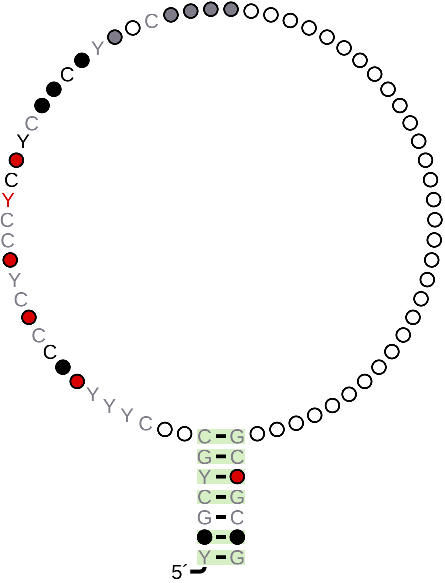

AR_M5_5U_1

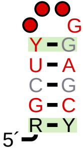

AR_M6_5U_1

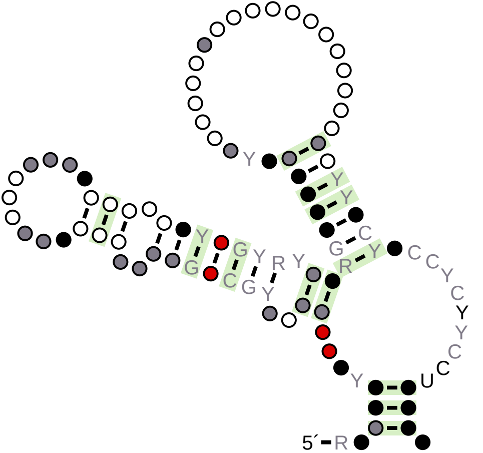

AR_M7_5U_1

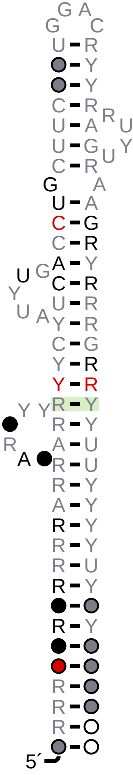

AR_M8_-1_5U_1

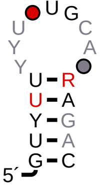

AR_M9_-1_5U_1

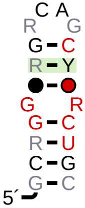

AR_M11_-1_5U_1

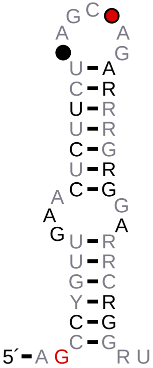

AR_M12_-1_5U_1

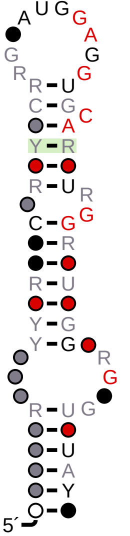

AR_M13_CDS_1

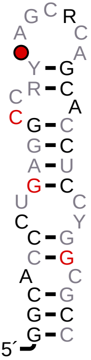

AR_M14_CDS_1

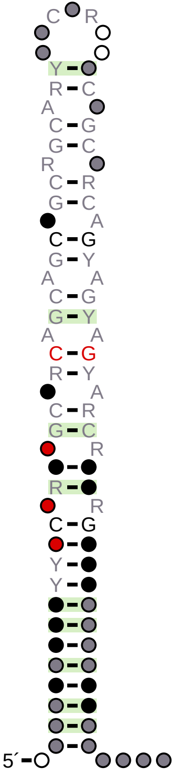

AR_M15_CDS_1

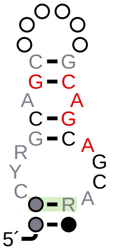

AR_M16_CDS_1

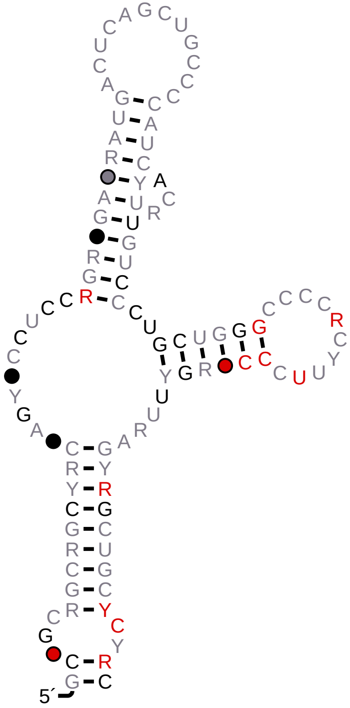

AR_M17_CDS_1

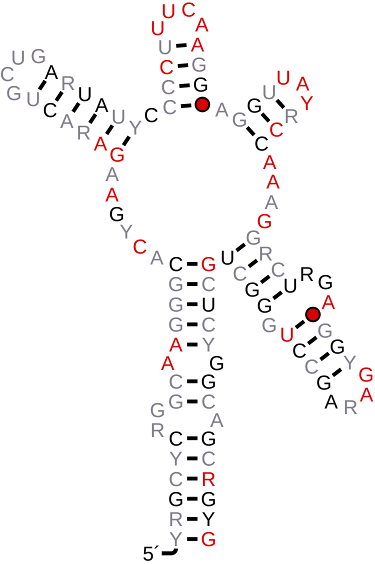

AR_M18_CDS_1

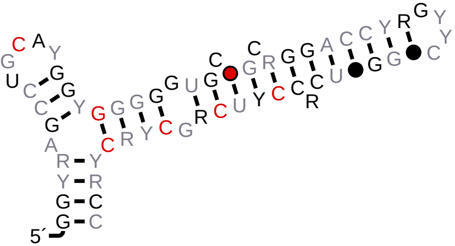

AR_M19_CDS_1

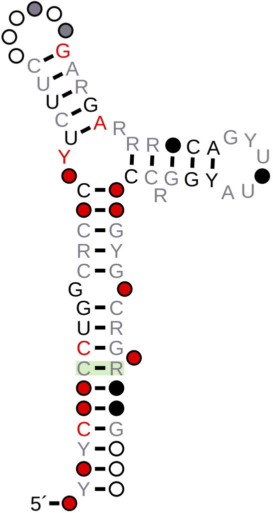

AR_M20_CDS_1

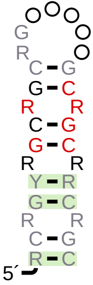

AR_M21_CDS_1

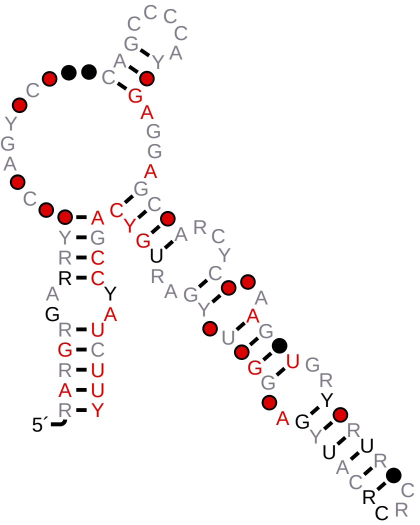

AR_M22_CDS_1

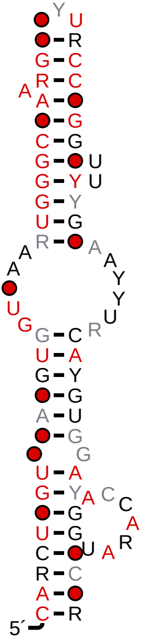

AR_M23_CDS_1

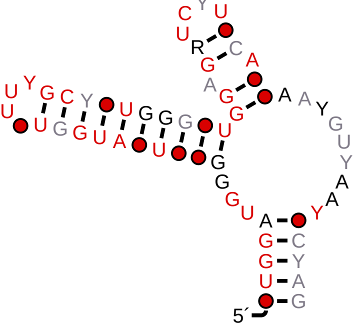

AR_M24_CDS_1

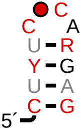

AR_M25_CDS_1

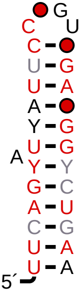

AR_M26_CDS_1

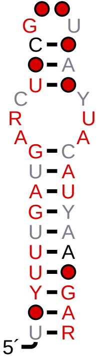

AR_M27_CDS_1

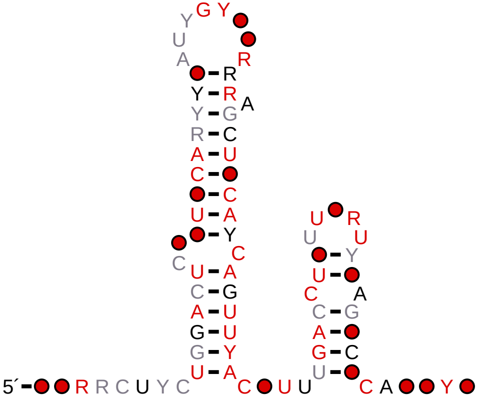

AR_M28_CDS_1

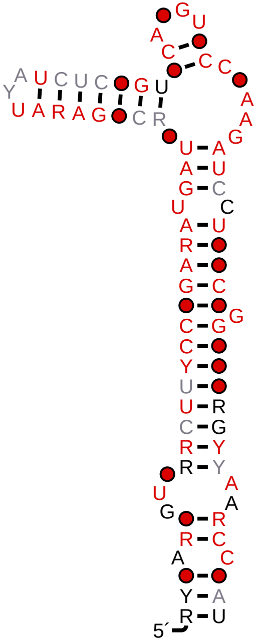

AR_M29_1_1

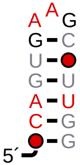

AR_30_-1_1

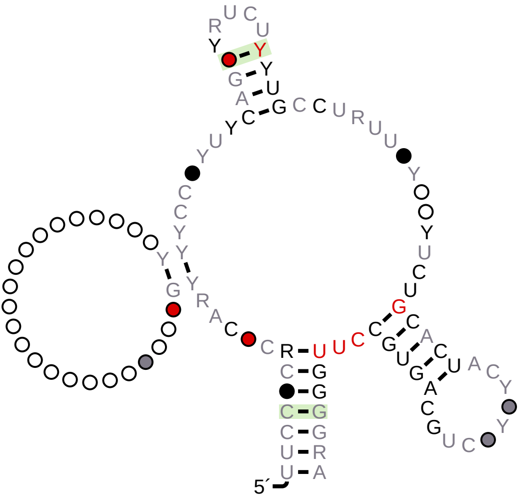

AR_M31_1

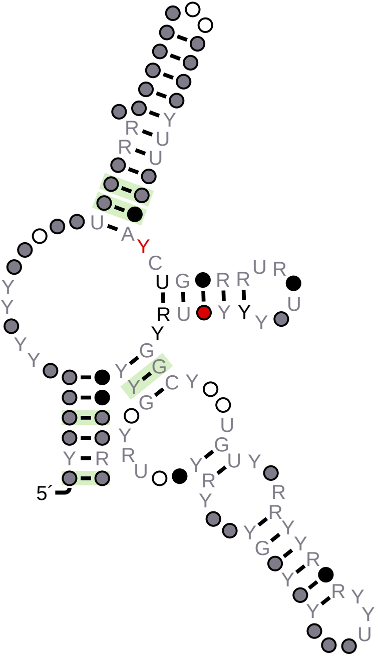

AR_M32_1

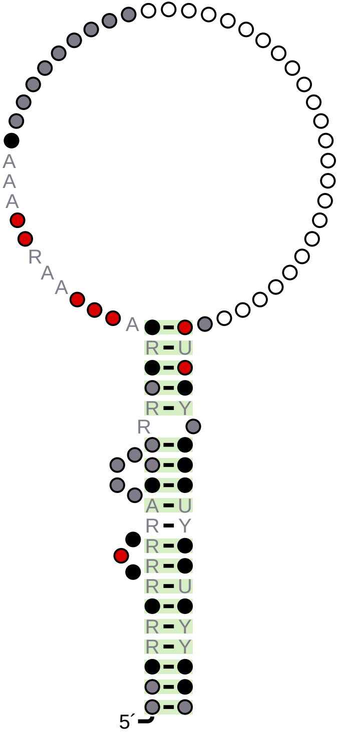

AR_M33_1

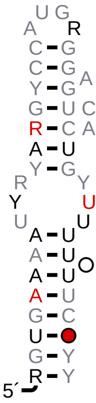

AR_M34_1

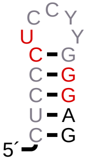

AR_M35_1

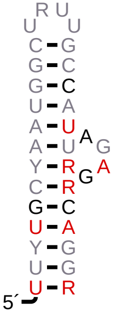

AR_M36_1

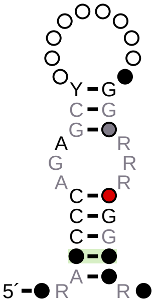

AR_M37_-1_1

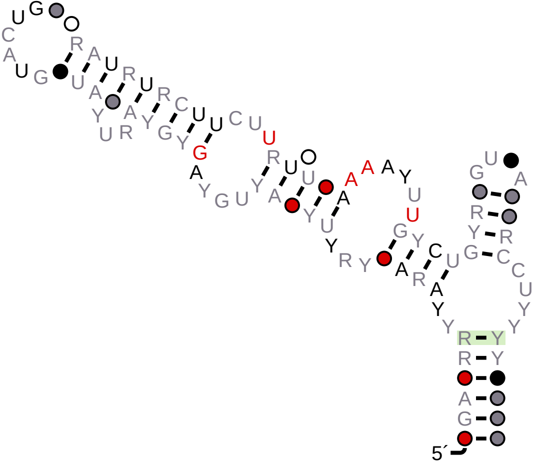

AR_M38_-1_1

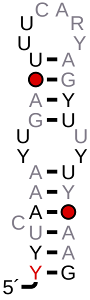

AR_M39_-1_1

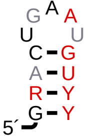

AR_M40_-1_1

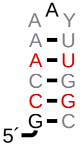

AR_M41_1

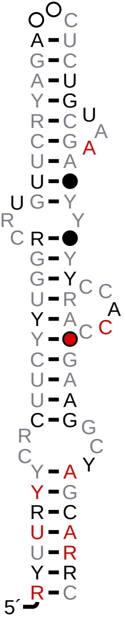

AR_M42_1

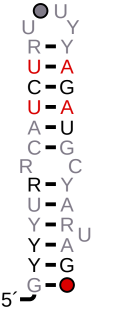

AR_M43_-1_1

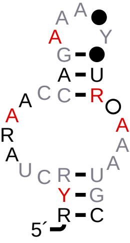

AR_M44_1

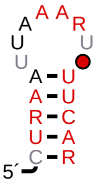

AR_M46_1

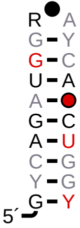

AR_M47_-1_1

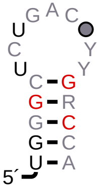

AR_M48_-1_1

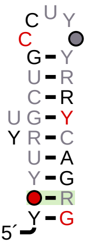

AR_M49_-1_1

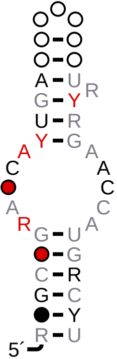

AR_M50_1

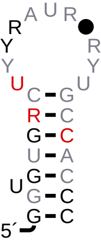

AR_M51_1

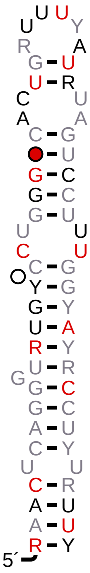

AR_M52_-1_1

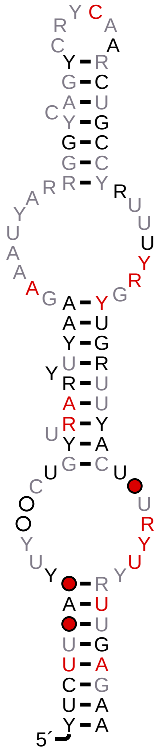

AR_M53_1

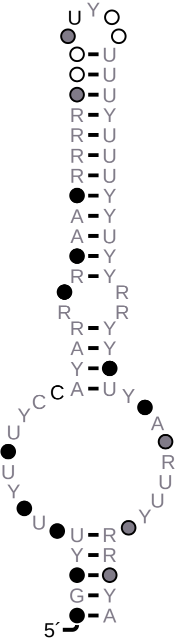

U Y
U
U
U
R Y
R U
R U
R U
Y
A Y
A U
Y
R Y
R
R
R
R Y
A Y
Y
C A U Y
Y C
A
U
R
U
U
Y
U
Y
U
U R
Y R
G Y
A
5´

AR_M55_1

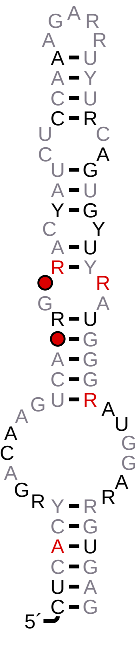

AR_M56_1

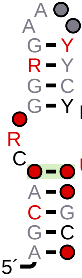

AR_M57_1

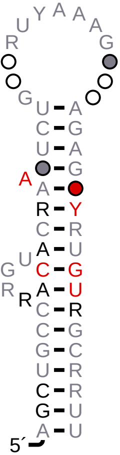

AR_M58_1

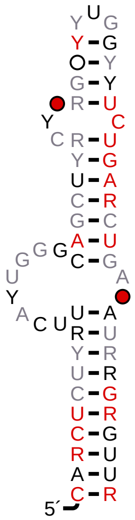

AR_M59_1

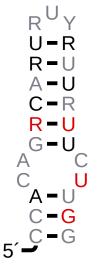

AR_M60_1

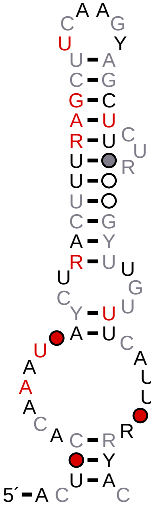

AR_M61_1

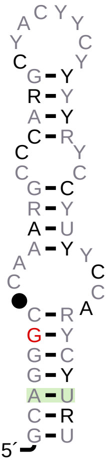

AR_M62_1

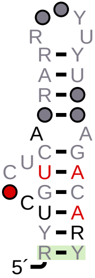

AR_M63_-1_1

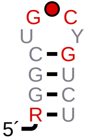

AR_M64_-1_1

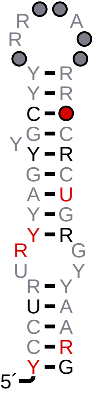

AR_M65_-1_1

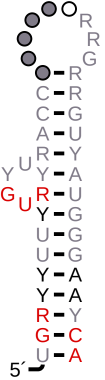

AR_M66_-1_1

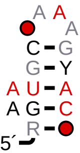

AR_M67_-1_1

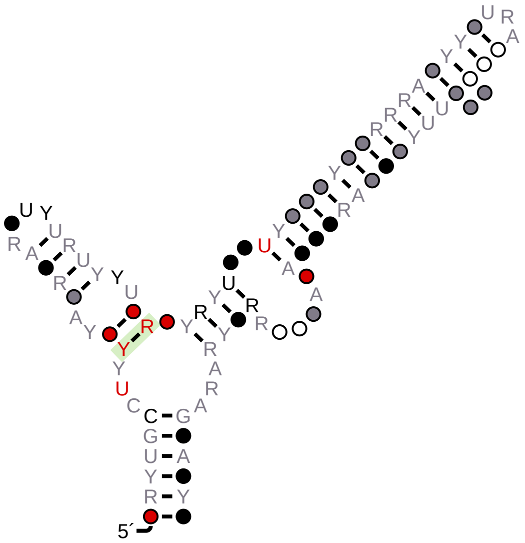

AR_M68_-1_1

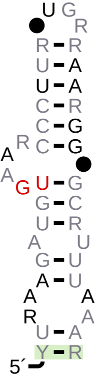

AR_M69_-1_1

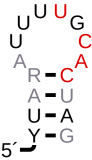

AR_M70_-1_1

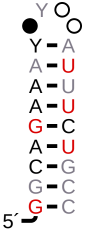

AR_M71_1

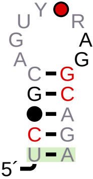

AR_M72_1

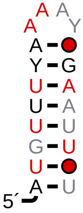

AR_M73_AURep_1

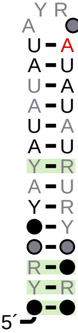

AR_M74_-1_1

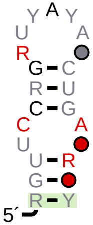

AR_M75_-1_1

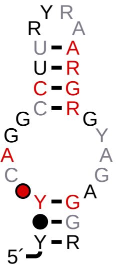

AR_M76_-1_1

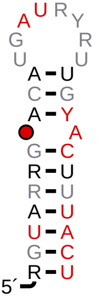

AR_M77_-1_1

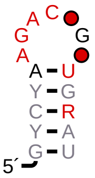

AR_M78_-1_1

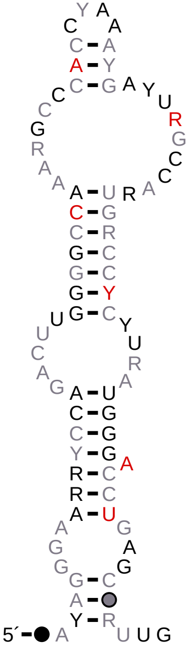

AR_M79_-1_1

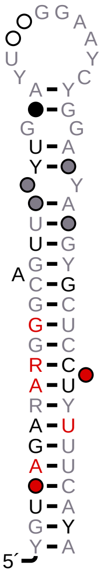

AR_M80_1

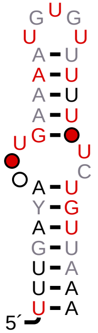

AR_M81_1

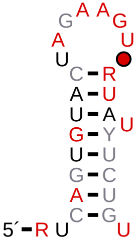

AR_M82_1

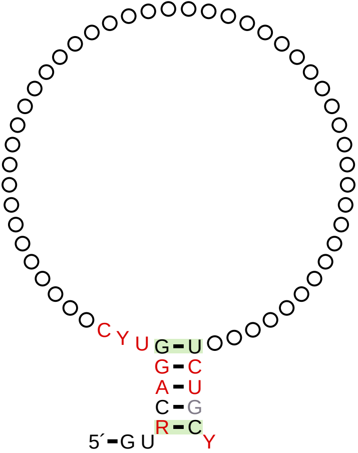

AR_M83_1

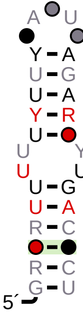

AR_M84_1

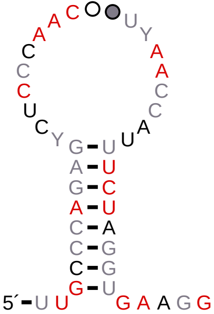

AR_M85_1

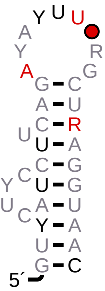

AR_M86_1

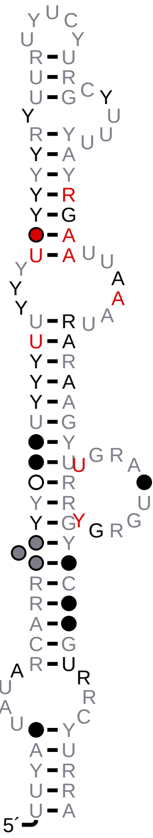

AR_M87_-1_1

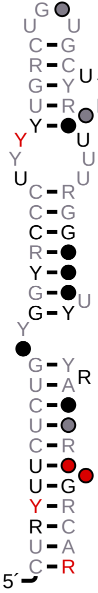

AR_M88_-1_1

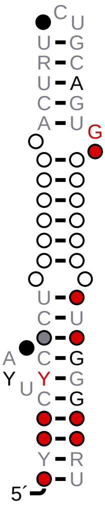

AR_M89_-1_1

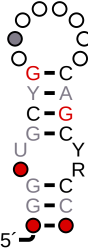

Supplement: gkae220_Supplemental_Files [file gkae220_supplemental_files.zip › Supplementary File S5.zip!/Supplementary File S5/FL_CaCo.zip!/FL_CaCo/AR_FL_CaCoFold.pdf]
